# *DLGAP5* mutations Disrupt Normal Chromosome Segregation and Spindle Formation of human Oocyte Meiosis and Lead to Female Infertility

**DOI:** 10.1002/mco2.70224

**Published:** 2025-05-22

**Authors:** Meng Wang, Zhou Li, Juepu Zhou, Rui Long, Qingsong Xi, Hong Gao, Youzhu Li, Lei Jin, Lixia Zhu

**Affiliations:** ^1^ Reproductive Medicine Center Tongji Hospital Tongji Medical College Huazhong University of Science and Technology Wuhan China; ^2^ Department of Reproductive Medicine The First Affiliated Hospital of Xiamen University School of Medicine, Xiamen University Xiamen China

1

Dear Editor,

Nowadays, approximately one out of six couples of reproductive age are confronted with infertility. Assisted reproductive technology (ART) constitutes one of the most effective measures to address this issue; however, in certain instances, ART fails [1]. Moreover, some patients experience recurrent ART failure with a uniform phenotype, suggesting a genetic component to their condition [2]. Recently, the advent of whole‐exome sequencing (WES) has led to the identification of an increasing number of genetic causes of human infertility [3]. However, the genetic underpinnings of a significant proportion of cases remain enigmatic [3]. Consequently, it is imperative to elucidate the potential genetic determinants of ART failure and to identify novel genetic etiologies for genetic counseling, as well as for the diagnosis and treatment of infertility patients.

In this study, we observed a pair of sisters who were undergoing ART. Both of the sisters were diagnosed with unexplained primary infertility. Following numerous in vitro fertilization (IVF) and/or intracytoplasmic sperm injection (ICSI) cycles, they failed to conceive, and the majority of their oocytes were immature. Subsequent genetic analysis identified a homozygous nonsense mutation c.1101C>G (p.Tyr367*) in *DLGAP5* in both sisters. The parents of the sisters are known to be from a consanguineous marriage family (family 1). The father was a heterozygous carrier, and the mother had already passed away (Figure 1A). This mutation is not indexed in the gnomAD (v4.1) database, and the affected amino acids were found to be highly evolutionarily conserved in different species. In the further expansion of screening, the same homozygous mutation c.1101C>G (p.Tyr367*) in *DLGAP5* was also identified in another patient suffering from primary infertility. Her mother was found to be a heterozygous carrier of the mutation (Figure 1A). She has undergone three fresh IVF cycles, and the majority of oocytes retrieved were also immature (Table S1). Consequently, no embryos were available, and the cycles had to be terminated.

**FIGURE 1:**
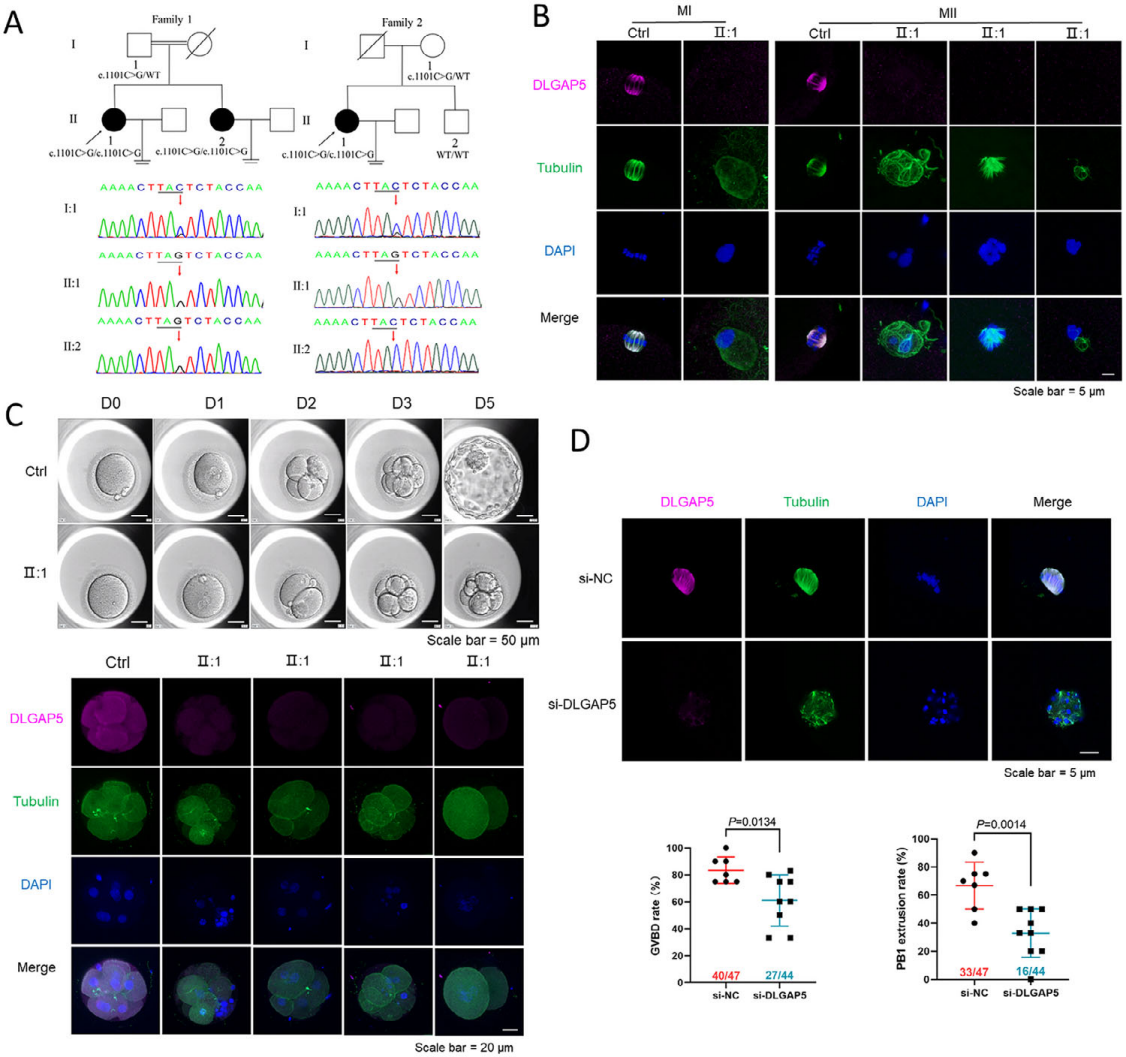
DLGAP5 mutations disrupt normal chromosome segregation of human oocyte meiosis and lead to female infertility **(A)** The pedigrees presented with abnormal oocyte development. In Family 1, the proband and her sibling sister were from a consanguineous family with a recessive inheritance pattern. In Family 2, the proband was a sporadic case. **(B)** Effects of *DLGAP5* mutation on spindle morphology and DLGAP5 level in oocytes. The expressions of DLGAP5 were absent with disorganized spindles and poorly aligned chromosomes in MI and MII oocytes of the proband. **(C)** Embryo development of the proband from family 1 under time‐lapse monitor system, and effects of *DLGAP5* mutation on the expression of DLGAP5 in embryos. The embryos of the proband cleaved slowly and arrested at D3. The signals of DLGAP5 staining in embryos of the proband were weaker than controls. **(D)** Effects of DLGAP5 depletion on DLGAP5 level and spindle morphology in human oocytes using immunofluorescence staining, and effects of *DLGAP5* depletion in human oocytes on GVBD rate at 24 h and PB1 extrusion rate at 48 h in IVM. MI, metaphase I; MII, metaphase II; Ctrl, control; NC, negative control; GVBD, germinal vesicle breakdown; PB1, polar body 1; IVM, in vitro maturation.

Discs large‐associated protein 5 (DLGAP5), also known as hepatoma upregulated protein (HURP), has been initially found to be upregulated in hepatocellular carcinoma cells [4]. It has been demonstrated to play a role in cell division and cancer biology [5]. Research has shown that DLGAP5 is a microtubule‐associated protein that contributes to chromosome movement and alignment by stabilizing mitotic microtubules and regulating microtubule dynamics to ensure proper chromosome segregation during cell division [6, 7, 8]. In addition, female *Dlgap5* knockout mice are infertile [9]. These animals have been observed to exhibit implantation failure due to defects in endometrial mesenchymal proliferation [9], as well as aneuploid embryos resulting from aberrant meiotic divisions of oocytes [10]. However, but the specific function of *DLGAP5* in human oocyte maturation and female fertility remains unclear. Utilizing WES, for the first time, we have identified *DLGAP5* as a pathogenic gene responsible for human female infertility with phenotype, characterized by abnormal oocyte maturation and embryo development.

In the ensuing exploration, it was ascertained that DLGAP5 exerts an influence on the process of oocyte meiosis by interfering with normal chromosome segregation. Immunofluorescence staining was performed to analyze the expression of DLGAP5. In the control group, DLGAP5 was found to be co‐localized with microtubules of the spindle in oocytes, and the structure of the spindles was well organized to ensure normal chromosome segregation from MI to MII transition. However, in the oocytes from proband of family 1, the expression of DLGAP5 was absent. The spindle morphology was found to be abnormal, and the chromosomes were poorly aligned, irrespective of the oocyte stage (MI or MII) (Figure 1B). In addition, although the MII oocytes can be fertilized successfully through IVF, the developmental potential of the embryos was impaired, with no cleavage embryos developing into blastocysts (Figure 1C). Immunofluorescence analysis revealed that the signals of DLGAP5 staining in embryos from the proband were weaker compared to those observed in the normal controls. Additionally, the analysis revealed the presence of fragmented nuclei in the blastomeres of the affected embryos (Figure 1C). Furthermore, whole‐embryo karyotype sequencing of three embryos from the proband of family 1 indicated abnormal karyotypes: 46, XN, ‐1(×1), +2(×3), +4p(p16.1→p12, ∼39Mb, ×3), ‐4q(×3), ‐9(×1, mos, ∼50%), 12p(p13.31→p11.21, ∼24Mb, ×1, mos, ∼54%), ‐12q(q11→q13.11, ∼10.0Mb, ×1, mos, ∼52%), +14(×3, mos, ∼53%), +16q(×3, mos, ∼51%), +17p(×3), +17q(q11.2→q24.2, ∼41Mb, ×3), +20(×3, mos, ∼58%); 45, Y, ‐X(×0), +Yq(q11.21→q11.221, ∼4.3Mb, ×2), ‐7p(p12.3→p11.2, ∼10Mb, ×1, mos, ∼51%); 46, XN, ‐2q(q21.2→q32.3, ∼63Mb, ×1, mos, ∼55%). This finding suggests the occurrence of an aberrant chromosome segregation during meiosis in oocytes from affected females.

To further validate the previous results, human GV oocytes were microinjected with *DLGAP5* siRNAs and then cultured in vitro. As shown in Figure 1D, the oocytes microinjected with si‐DLGAP5 exhibited a significantly reduced GVBD rate (*p* = 0.0134) at 24 h and a lower PB1 extrusion rate (*p* = 0.0014) at 48 h. Moreover, the expression of DLGAP5 was significantly reduced in MII oocytes in the *DLGAP5* siRNA injection group compared to those injected with negative controls. Meanwhile, the typical morphology of the spindle microtubules was absent with the disarrangement of the chromosomes.

In this study, we identified a novel homozygous mutation c.1101C>G, p.Tyr367* in *DLGAP5* by WES, and *DLGAP5* is proposed as a causal gene related to oocyte meiosis disorder for the first time. During meiosis, proper spindle assembly is essential for oocyte development and accurate chromosome segregation. Previous studies have shown that DLGAP5 plays a significant role in the organization of the microtubule organizing center and the formation of the oocyte spindle in animal models [10]. Some studies have reported that DLGAP5 is involved in the regulatory processes of cyclization changes in the endothelium [9]. Nevertheless, none of these studies have investigated the function of DLGAP5 in human oocytes or included samples from patients with DLGAP5 mutations. In the present study, we have confirmed for the first time the important role of DLGAP5 in human oocyte meiotic progression. The nonsense mutation in *DLGAP5* resulted in abnormal oocyte maturation and embryo development. Furthermore, the reduced expression of DLGAP5 in human oocytes by microinjection of siRNA targeting DLGAP5 resulted in delayed maturation in vitro. Similarly, in IVM oocytes, the spindle morphology was abnormal and the chromosome arrangement was disordered, suggesting that DLGAP5 depletion could impair chromosome segregation in oocytes and thus lead to an abnormal karyotype in subsequent embryos. The results of the whole‐embryo karyotype sequencing in our study confirmed this conclusion. These results indicate that DLGAP5 is essential for meiotic spindle assembly and highlight the importance of maintaining regular DLGAP5 expression and function in oocyte meiosis and embryo development.

In conclusion, we have identified a novel homozygous nonsense *DLGAP5* mutation c.1101C>G, p.Tyr367* in infertile females characterized by abnormal oocyte maturation and embryo development. Our study expanded the current spectrum of pathogenic genes responsible for the phenotype of oocyte maturation and embryo development defects. This study provided a theoretical basis and application values for clinical counseling, genetic diagnosis, and treatment strategies in infertile patients.

## Author Contributions

**Zhou Li, Youzhu Li, Lei Jin, and Lixia Zhu**: designed the experiment. **Meng Wang, Juepu Zhou, and Rui Long**: conducted experiments. **Qingsong Xi and Hong Gao**: collected samples. **Meng Wang**: analyzed the data and drafted the manuscript. **Lixia Zhu**: revised the manuscript. All authors have read and approved the final manuscript.

## Ethics Statement

This study was approved by the Ethical Committee of Tongji Hospital (TJ‐IRB20200722). Signed informed consent and information use forms were obtained from patients. All the oocytes and embryos investigated in this research from the affected individuals were donated with written informed consent.

## Conflicts of Interest

The authors have no interest to disclose.

## Supporting information



Supporting Information

## Data Availability

The datasets supporting the current study are available from the corresponding authors upon request.
