# Supplementary material for: DLGAP5 mutations Disrupt Normal Chromosome Segregation and Spindle Formation of human Oocyte Meiosis and Lead to Female Infertility
Source: MedComm (2020). 2025 May 22;6(6):e70224. doi: 10.1002/mco2.70224 (PMC12099062; doi:10.1002/mco2.70224)
Supplement: Supplementary file 1 — Supporting Information [file MCO2-6-e70224-s001.docx]

**Material and methods**

**Subjects and genetic studies**

Patients undergoing *in vitro* fertilization (IVF)/ intracytoplasmic sperm injection (ICSI) treatment were enrolled. When three dominant follicles with a diameter larger than 18 mm were observed after a standard controlled ovarian hyperstimulation, 10,000 IU human chorionic gonadotropin (HCG) was administered. Whole-exome sequencing (WES) was recommended for patients with oocyte maturation arrest, which was identified by the majority of retrieved oocytes being immature at the germinal vesicle (GV) and/or metaphase I (MI) stage. Peripheral blood was collected from patients and their family members, and genomic DNA was extracted. Whole exomes were captured, and sequencing was performed on the Illumina HiSeq X-TEN platform (Illumina, San Diego, USA). Raw FASTQ files were mapped to the human reference sequence (hg19/GRCh37). Gene mutations were annotated using the dbSNP, 1000 Genomes, and ExAC databases. Functional predictions were based on REVEL, a combination of databases, including the SIFT, PolyPhen-2, and MutationTaster. Candidate mutations meeting the following inclusion criteria were considered for further analysis: a. with a frequency < 0.1% for homozygous mutations or < 1% for compound heterozygous mutations in population frequency databases (including gnomAD, 1000 Genomes, ChinaMap, and our local population databases of over 100,000 individuals); b. synonymous variants without splicing effects predicted by spliceAI MaxEntScan software were removed; c. missense variants predicted to be benign or likely benign by protein function prediction software (including SIFT, Polyphen2_HDIV, Polyphen2_HVAR, and MutationTaster) were removed; d. variant sites of exonic region or splicing site region (±20) were retained; e. homozygous or compound heterozygous variant sites were retained; f. gene function annotation to find genes previously reported to affect or be likely to affect female fertility in humans or mice. Candidate genes and mutations were confirmed by Sanger sequencing of the candidate patients and their family members.

***In vitro* maturation and microinjection of human GV oocytes**

Human germinal vesicle (GV) oocytes were obtained from patients at our center who donated for research with written informed consent. Oocyte donors were selected from patients undergoing ICSI for male-factor infertility, aged from 25 to 35 years with normal ovarian reserve. Moreover, any conditions known to affect oocyte maturation and development were excluded. The oocytes retrieved were of normal morphology, and the results of ICSI outcomes were also normal. A small interference RNA (siRNA) targeting *DLGAP5* was microinjected into GV oocytes, and cultured for 24 h for endogenous *DLGAP5* knockdown in a G1-plus medium (Vitrolife, Sweden) supplemented with milrinone (Cat: HY-14252, MCE, Shanghai, China) in a time-lapse incubator with 6% CO_2_ and 5% O_2_ at 37 ℃. After release, GV oocytes were subsequently cultured for *in vitro* maturation. The GV breakdown (GVBD) and polar body 1 (PB1) extrusion were recorded after release. The time from GVBD to PB1 extrusion was also calculated. The *DLGAP5* siRNAs were purchased from GenePharma, Shanghai, China (si-1, 5’-AGACUAAGAUUGAUAACGA-3’; si-2, 5’-AGACUAAGAUUGAUAACGA-3’; si-3, 5’-GGUCUAAACUGCAGUAAUC-3’; si-4, 5’-UAAAGUGGGUCGUUAUAGA-3’).

**Immunofluorescence staining**

Oocytes and embryos were fixed in 4% paraformaldehyde and stained. For spindle and chromosome imaging, Tubulin-Tracker Green (1:100 dilution, Cat: C1051S, Beyotime, Shanghai, China) was used for the spindle localization and DAPI (Cat: G1012, Servicebio, Wuhan, China) for DNA visualization. A normal spindle is characterized by its spindle-shaped morphology and symmetry, with a prominent pole on each side. In contrast, the abnormal spindle has a disintegrated structure, resulting in a shortened length. The normal chromosome is aggregated and aligned in an orderly manner on the equatorial plate, whereas the abnormal chromosome may separate from the equatorial plate, characterized by excessive looseness or a scattered distribution. For the other immunofluorescence staining, the primary antibody was used at 1:100 dilution. Cy3-conjugated secondary antibody was used at 1:200. The detailed information of the primary antibody included: anti-DLGAP5 antibody (Cat: A13575, Abclonal, Wuhan, China). Images were captured with an LSM900 confocal microscope (Leica, Germany).

**Table S1 Clinical characteristics and IVF/ICSI outcomes of the affected individuals**

| **Patient ID** | **Age (years)** | **Duration of infertility (years)** | **IVF and ICSI cycles** | **COH protocol** | **Total No. of oocytes retrieved** | **GV oocytes** | **MI oocytes** | **MII oocytes** | **Fertilized oocytes** | **No. of available embryos** |
| --- | --- | --- | --- | --- | --- | --- | --- | --- | --- | --- |
| Family 1 II:1 | 35 | 6 | 1/1 | GnRH agonist | 22 | 5 | 16 | 1+9* | 6 | 0 |
| Family 1 II:2 | 30 | 9 | 1/1 | GnRH agonist | 18 | 1 | 17 | 0+4* | 0 | 0 |
| Family 2 II:1 | 31 | 4 | 1/3 | GnRH agonist | 11 | 2 | 9 | 0 | 0 | 0 |
|  |  |  | 2/3 | GnRH antagonist | 12 | 3 | 6 | 3 | 2 | 0 |
|  |  |  | 3/3 | PPOS | 16 | 2 | 12 | 2 | 2 | 0 |

Notes:

* MII oocyte originated from immature oocytes after one-day *in vitro* maturation.

IVF, in vitro fertilization; ICSI, intracytoplasmic sperm injection; COH, controlled ovarian hyperstimulation; GV, germinal vesicle; MI, metaphase I; MII metaphase II; GnRH, gonadotrophin-releasing hormone; PPOS, progestin primed ovarian stimulation.
